# The composition and initial evaluation of a grimace scale in ferrets after surgical implantation of a telemetry probe

**DOI:** 10.1371/journal.pone.0187986

**Published:** 2017-11-13

**Authors:** Marsinah L. Reijgwart, Nico J. Schoemaker, Riccardo Pascuzzo, Matthew C. Leach, Melanie Stodel, Loes de Nies, Coenraad F. M. Hendriksen, Miriam van der Meer, Claudia M. Vinke, Yvonne R. A. van Zeeland

**Affiliations:** 1 Animal Research Centre, Institute for Translational Vaccinology (Intravacc), Bilthoven, The Netherlands; 2 Department of Animals in Science & Society, Faculty of Veterinary Medicine, Utrecht University, Utrecht, The Netherlands; 3 Department of Clinical Sciences of Companion Animals, Division of Zoological Medicine, Faculty of Veterinary Medicine, Utrecht University, Utrecht, The Netherlands; 4 Department of Mathematics, MOX Laboratory for Modelling and Scientific Computing, Politecnico di Milano, Milan, Italy; 5 School of Agriculture, Food & Rural Development, Newcastle University, Newcastle upon Tyne, United Kingdom; University of Bari, ITALY

## Abstract

Reliable recognition of pain is difficult in ferrets as many currently available parameters are non-specific, inconsistent and/or impractical. Grimace scales have successfully been applied to assess pain in different animal species and might also be applicable to ferrets. To compose a Ferret Grimace Scale (FGS), we studied the facial musculature of ferrets and compared lateral photographs of 19 ferret faces at six time points before and after intraperitoneal telemetry probe implantation. We identified the Action Units (AUs) *orbital tightening*, *nose bulging*, *cheek bulging*, *ear changes* and *whisker retraction* as potential indicators of pain in ferrets. To evaluate whether these AUs could reliably be used to identify photographs taken before and after surgery, the photographs were scored 0, 1 or 2 (not, moderately or obviously present) by 11 observers that were blinded to the treatment and timing of the photographs. All AU-scores assigned to the photographs taken five hours after surgery were significantly higher compared to their time-matched baseline scores. Further analysis using the weights that were obtained using a Linear Discriminant Analysis revealed that scoring *orbital tightening* alone was sufficient to make this distinction with high sensitivity, specificity and accuracy. Including weighted scores for *nose bulging*, *cheek bulging* and *ear change* did not change this. As these AUs had more missing values than *orbital tightening*, their descriptions should be re-evaluated. Including *whisker retraction*, which had a negative weight, resulted in lower accuracy and should therefore in its current form be left out of the FGS. Overall, the results of this study suggest that the FGS and the AU *orbital tightening* in particular could be useful in a multifactorial pain assessment protocol for ferrets. However, before applying the FGS in practice, it should be further validated by incorporating more time points before and after applying (different) painful stimuli, and different levels of analgesia.

## Introduction

Ferrets (*Mustela putorius furo*) are routinely used as an animal model of human disease (e.g. influenza), during which these animals often undergo a disease process associated with a high risk of (unrelieved) pain (note: in this article, pain will be used as a definition including pain, discomfort, distress and suffering, as these definitions overlap and are difficult to measure in animals [1]). To optimize animal welfare during these studies, refinement strategies should be implemented, such as timely and accurate recognition and treatment of pain. Unfortunately, scientific data on the assessment of pain in ferrets is scarce, complicating the recognition of pain in these animals [2].

The latest approach in the assessment of pain in animals are the species-specific grimace scales that appear to utilize our tendency to focus on the face of an animal [3]. Also, grimace scales can easily and rapidly be taught to animal caretakers [4,5] and appear to be less time consuming than other pain assessment methods, e.g. activity monitoring [5]. Moreover, live-scoring in a laboratory setting has been suggested to be practically applicable [6].

Grimace scales are based on the Facial Action Coding System (FACS) for humans, which describes changes to the surface appearance of the face using action units (AUs) such as brow lowering, tightening and closing of the eye lids, nose wrinkling and upper lip raising [7,8]. The FACS has been used successfully to assess pain in humans that are unable to communicate with their clinicians (e.g. people with cognitive impairment and neonates) [9,10]. The first grimace scale that was developed and validated for laboratory animals was the mouse grimace scale [11]. This was followed by the development of grimace scales for rats [12], rabbits [13], horses [14,15], cats [16], cattle [17], sheep [18], piglets [19] and lambs [20]. To the authors’ knowledge, a grimace scale for ferrets has not yet been developed. The current grimace scales share changes in a number of the similar AUs that correspond to changes in the shape and/or position of the eyelids, cheek, nose, whiskers and ears, which also correspond with those observed to change in humans when in pain [21]. This similarity suggests evolutionary conservation of key expressions of pain [22], which led to the hypothesis that ferrets would exhibit similar visible facial changes when experiencing pain, provided they possess the facial musculature to do so.

Understanding which muscles could be involved in facial expressions associated with pain could aid in identification of AUs and interspecies grimace scale comparisons. As no anatomical illustrations of the facial musculature of ferrets are readily available and ferrets are anecdotally said to lack the muscles to express the AUs seen in other species, an anatomical study was performed to document the different facial muscles in ferrets. Subsequently a Ferret Grimace Scale (FGS) with five AUs was composed, based on the information provided by the anatomical study and by using the grimace scales of other species. The clarity of the AU-descriptions as well as the sensitivity, specificity, and accuracy was assessed. Photographs were taken of ferrets’ faces before and after undergoing a moderate to severe pain stimulus (i.e. surgical implantation of an intraperitoneal telemetry probe without subsequent analgesics) to see if double-blind observers could differentiate pre- and post-surgery photographs.

## Animals, materials & methods

### Ethical note

This study was carried out in accordance with the European legislation for the protection of animals used for scientific purposes (Directive 2010/63/EU) [23] and ethically approved by the Institutional Animal Care and Use Committee (IACUC) of Intravacc (DEC201200260). The authors were kindly allowed to observe and photograph the ferrets that were undergoing a surgical procedure as part of this unrelated influenza study.

### Facial musculature

To study the anatomy of the facial muscles, three humanely euthanized ferrets (from DEC201400137) were used to document the facial musculature. Dissection was started with a surgical cut through the skin layer in caudo-rostral direction. For proper investigation of the superficial musculature, the skin layer was removed in direction of the eye and muzzle at one lateral side. This was followed by the removal of the superficial muscles to further investigate the deep musculature. Methods of dissection were the same for both sides of the face. The facial muscles were compared to detailed anatomical drawings of the facial musculature of the cat, dog and rodents [22] and a professional veterinary anatomic illustrator (R. van Deijk) composed a detailed anatomical drawing of the ferrets’ facial muscles.

### Animals and husbandry

To compose and evaluate the FGS, we used 19 domestic intact female ferrets of different coat colour and length (12 regular sables, 3 angora-like sables, 4 regular black-eyed whites). The ferrets were 16–32 weeks old, weighed 783±86 grams (range: 660–940 grams), and were obtained from Schimmel BV, The Netherlands. The ferrets were group-housed indoors in open phenolic face plywood ground cages (94.5x 166 x 64.6 cm) on wood shavings (IRS LIGNOCELL® Hygienic animal bedding). Ferrets were maintained on a 8:16 hour light:dark cycle (lights on from 8.00AM to 16.00PM) at 18–25°C. Auditory stimulation in the form of a radio was present 24 hours/day. Food (Hope farms® Ferret balance pellets) and water were given *ad libitum* in stoneware bowls. Cage enrichment consisted of a ferret ball (25 cm diameter with 6 holes of 7.5 cm each) and a large, 24L flexible sleeping bucket. The animals were acclimated to their housing conditions for 27 days prior to the surgery. The ferrets were weighed weekly and their health was monitored daily.

### Surgery and recovery

Prior to anaesthesia a physical examination was performed on the ferrets to ascertain that they were in good health. Anaesthesia was induced by administering 0.2 ml of a 9:1 mixture of ketamine (Narketan, 100 mg/ml, range: 16–26 mg/kg)–dexmedetomidine (Dexdomitor®, 0.5 mg/ml, range 8–12 μg/kg) in the left caudal thigh musculature. The ferrets were placed in a dorsal recumbence and the ventral aspect was shaved and disinfected with 70% alcohol. A midline incision, with a maximum length of two centimetres, was made in between the most cranial nipples, through the skin, *linea alba* and peritoneum to enter the abdomen. The time of incision was registered as T_0_. Two telemetry probes (DST micro-T and -HRT, STAR ODDI, 8.3 mm diameter, 25.5 long) were inserted into the abdomen. The *linea alba* and skin were separately closed with interrupted sutures (Vicryl® 3–0, Ethicon). All surgeries were performed by experienced technicians who were able to perform the surgery procedure on a single animal within 15 minutes, thereby enabling all surgeries to be completed between 9:00 and 11:30 AM of the same day. Because of the short duration of the surgery, no additional heat support was needed to allow the ferrets to maintain their body temperature. Following surgery, a single intra-muscular injection of 0.02 ml Antisedan® (Atipamezole, 5 mg/ml) REG NL 7744, average: 121 μg/kg, range: 92–145 μg/kg) was given in the right caudal thigh area to reverse the anaesthesia. The ferrets were then returned to their group-housing for recovery, where the water bowl was temporarily removed for safety reasons. The ferrets were continuously monitored until they were fully awake, at this time the water bowl was returned. Slowly recovering animals were placed in a separate cage with extra towels and a warm-water bottle wrapped in a towel for thermoregulation. As part of the influenza study protocol and with approval of the IACUC of Intravacc no post-surgical antibiotics or analgesics and other pain-relieving medication were administered.

### Photographs

In order to take photographs, the ferrets were individually taken out of their home cage and carried to an adjacent room. The ferret was then placed on a table in front of a PVC tube (75 mm diameter, 30 cm length), through which the ferret had to walk. For five days prior to the surgery the ferrets were individually habituated to this procedure, once per day for a period of 5 minutes.

**Fig 1:**
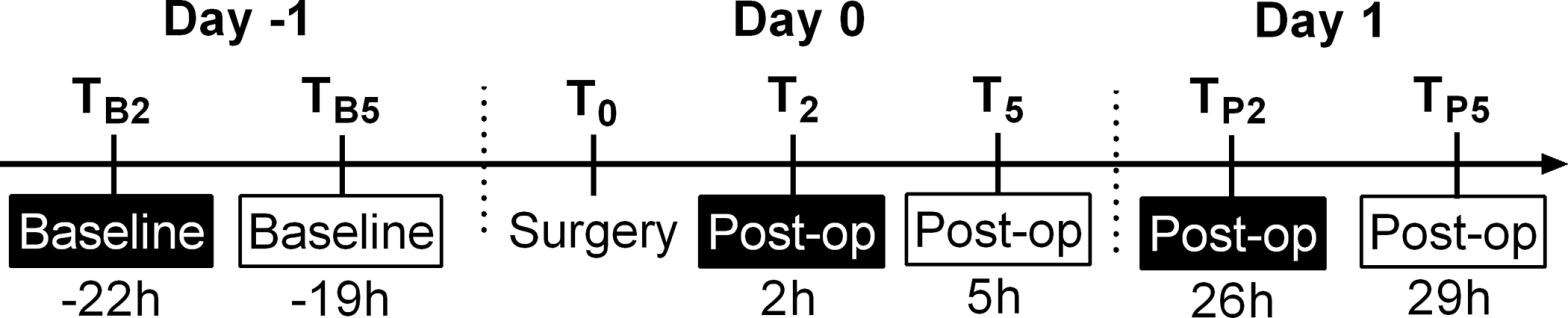
Timeline of the study. The photographs were taken at the time points in boxes. The black and white boxes are time-matched (T_B2_ was used as a baseline for T_2_ and T_P2_, while T_B5_ was used as a baseline for T_5_ and T_P5_).

Based on the anaesthesia recovery time [24,25] and grimace scale studies in other animal species (e.g. rats [12]), photographs were taken when the ferrets were fully recovered from the anaesthesia (i.e. 2 hours after surgery, T_2_) and when they were expected to show the most obvious grimace (as was seen in rats [12]) (i.e. 5 hours after surgery, T_5_). Baseline photographs were taken at time-matched moments on the day prior to surgery (i.e. 22 and 19 hours prior to surgery, T_B2_ and T_B5_). Additionally, time-matched photographs were taken on the day after surgery (i.e. 26 and 29 hours post-surgery, T_P2_ and T_P5_) (Fig 1).

The photographs were taken with a camera (Canon EOS 7D Mark II Body + Canon EF 50 mm 1.8 f II objective) that was placed lateral to the table at 58 cm distance from the end of the tube on a tripod. When the ferrets’ head was just outside of the tube, lateral photographs of the ferrets’ head were taken in burst mode (10 frames/second, 1/250, F = 5.0, ISO 10.000). This was repeated for the other side of the face. In total, each ferret walked through the tube 2–6 times per time point, depending on the suitability of the photographs taken. Selection of the photographs for further analysis was based on full visibility of the eyes, ears and whiskers. In addition, the position of the head needed to show the least angled (both horizontal and vertical). The selection of the photographs was performed blinded for time point and before identifying AUs to prevent selection bias. Selected photographs were cropped to contain only the head of the ferret and edited to contain a neutral background using Adobe Photoshop CC^®^.

### Composition of the Ferret Grimace Scale

Time-point blind comparisons were made between individual baseline photographs from each animal and the matching post-surgery photographs (T_B5_ vs T_5_) (within-subject comparison). Changes in facial expression within each ferret were recorded. Changes that were observed in five or more ferrets (>25%) were listed as action units (AUs). All AUs were listed in a Ferret Grimace Scale (FGS), designed to be scored on a three-point scale that is generally used for grimace scales (e.g. [11–13]). A score of ‘0’ indicated that the change in the AU was not present, a score of ‘1’ indicated that the change in the AU was moderately present and a score of ‘2’ indicated that the change in the AU was obviously present. Additionally, a short training module was composed for each AU, which showed three example photographs of each score (i.e. 0, 1 and 2) with lines indicating the changes visible for the AU.

### Evaluation of the Ferret Grimace Scale

To evaluate whether each of the five selected AUs could be used to differentiate ferrets before and after surgery, a survey was designed in Survey Monkey® and distributed to eleven observers. These observers included three ferret veterinarians, five ferret owners, one ferret researcher and two members of a ferret society/rescue that were blinded to the study objectives and time points. Each observer was asked to complete a 7-part survey, with each survey part sent to the observer one week after completion of the previous part.

Five of these survey parts (i.e. part 2–6) dealt with the individual AUs. In each of these, the observers were asked to rate each photograph (*N* = 114: 19 ferrets, 6 time points, i.e. 1 photograph per ferret per time point) on the three-point scale (0 = not present, 1 = moderately present, 2 = obviously present or? = I don’t know), after having completed a short training module for that specific AU. The order in which the AUs and the photographs were scored was randomized between observers (the order of the AUs was randomized using a balanced Latin square; the order of the photographs was randomized automatically by Survey Monkey®; Table 1).

**Table 1:** Time schedule of the 7-part survey with the scores that were assigned to the photographs of the ferrets. AU1-5 = action unit 1–5 of the Ferret Grimace Scale (*orbital tightening*, *nose bulging*, *cheek bulging*, *ear changes* and *whisker retraction*), presented in random order. Pre-training = intuitive overall pain assessment, post-training = overall pain assessment after being trained on the Ferret Grimace Scale.

Part 1	Part 2–6					Part 7
Pre-training	Ferret Grimace Scale					Post-training
Overall pain	**AU1**	**AU2**	**AU3**	**AU4**	**AU5**	**Overall pain**
0 = no pain 1 = minor pain 2 = moderate pain 3 = severe pain	0 = not present 1 = moderately present 2 = obviously present					0 = no pain 1 = minor pain 2 = moderate pain 3 = severe pain

Two survey parts (i.e. parts 1 and 7) comprised questions regarding the general assessment of pain. In these survey parts, the observers were asked to rate each photograph on the overall pain that the observer estimated the ferret to be in, using a subjective scoring method from an overall discomfort assessment scale used during animal experiments (0 = no pain, 1 = mild pain, 2 = moderate pain, 3 = severe pain or? = I don’t know). The scores from the first part (overall pain score) are the ‘pre-training’ scores, as the observers are not (yet) familiar with the AUs and have to rely on their own experience to assign a score. The scores from the last part (also overall pain score) are the ‘post-training’ scores, as the observers completed the survey part and the accompanying training for each AU (*orbital tightening*, *nose bulging*, *cheek bulging*, *ear changes* and *whisker retraction*)(Table 1).

### Statistical analysis

All analyses were performed using R (R core team, R Foundation for Statistical Computing). Packages are listed between brackets for each analysis. Differences were considered statistically significant if *P*<0.05. The p-values were corrected for multiple comparisons using the False Discovery Rate (FDR).

First, to get an indication whether the descriptions of the AUs were clear and whether training aided in assigning an overall score to the photographs, the percentage of missing data (scored with “I don’t know”) per AU per time point $\left(=\frac{\sum missing\;data\;{observer}_{1-11}\;on\;T_{x}}{209\;(11\;observers\times 19\;ferrets)}\times 100\right)$ and per ferret $\left(=\frac{\sum missing\;data\;{ferret}_{x}\;on\;T_{(-22)-29}\;for\;{AU}_{1-5}}{30\;(6\;timepoints\times 5\;AUs)}\times 100\right)$ were calculated. In addition, to further determine whether the descriptions of the AUs were clear and whether training aided in the agreement on the overall pain assessment, the inter-observer agreement was calculated for each AU and for the overall pain scores and the intra-observer agreement was calculated for the overall pain scores using an Intraclass Correlation Coefficient (ICC, “irr” package) [26]. A score of <0.40 is regarded as poor agreement, 0.40–0.59 as fair agreement, 0.6–0.74 as good agreement and 0.75–1.00 as excellent agreement [27].

Second, to assess whether the AUs could be used to differentiate photographs of ferrets’ faces before and after surgery, a Cumulative Link Mixed Model (CLMM, “ordinal” package) [28] was performed to assess how the time-point, the observer and the ferret influenced the score for each AU. This method is analogous to a linear mixed model, but specific to ordinal response values. The CLMM was performed with time point as a fixed effect and observer and ferret as random effects. The baseline scores (T_B2_ and T_B5_) were compared with each other and with the time-matched post-surgery (T_B2_ was compared to T_2_ and T_P2_, T_B5_ was compared to T_5_ and T_P5)_.

Third, the importance of each AU in discriminating the pain condition of a ferret was measured using a Linear Discriminant Analysis (LDA, “MASS” package) [29]. To quantitatively assess this importance, LDA assigns a weight to each AU, with the most important AUs having higher weights with respect to the others. If the weights are all equal, the linear weighted combination of the five AU scores is the mean of the scores. However, the weights are often not equal and the linear weighted combination of the five AU scores were used to maximize the discrimination between the photographs taken five hours after surgery (T_5_) and the corresponding baseline photographs (T_B5_). The AUs that were assigned a negative or near-to-zero weight were removed from the analysis and another LDA was performed. Using the optimal weights of the AUs obtained by performing the LDA, the sensitivity, specificity and accuracy with which the AUs could be used to discriminate photographs taken five hours after surgery (T_5_) from the corresponding baseline photographs (T_B5_) were calculated. The sensitivity (i.e. true positive rate) is the proportion of photographs taken at T_5_ (i.e. five hours after surgery) that were assigned a high score for the AUs properly weighed by the LDA weights $\left(\frac{T_{5}\;scored\;high\;according\;to\;LDA}{19\;(all\;T_{5}\;pictures)}\right)$. The specificity (i.e. true negative rate) is the proportion of photographs taken at T_B5_ (i.e. 19 hours before surgery) that were assigned a low score for the AUs properly weighed by the LDA weights $\left(\frac{T_{B5}\;scored\;low\;according\;to\;LDA}{19\;(all\;T_{B5}\;pictures)}\right)$. The accuracy (i.e. the ratio of the sum of true positives and true negatives, to the total number of correct or incorrect classifications) is the proportion of photographs taken at T_B5_ and T_5_ that were assigned a low and high score, respectively, properly weighed by the LDA weights $\left(\frac{T_{5}\;scored\;high\;according\;to\;LDA+T_{B5}\;scored\;low\;according\;to\;LDA}{38\;(all\;T_{5}+all\;T_{B5}\;pictures)}\right)$.

## Results

### Facial musculature

**Fig 2:**
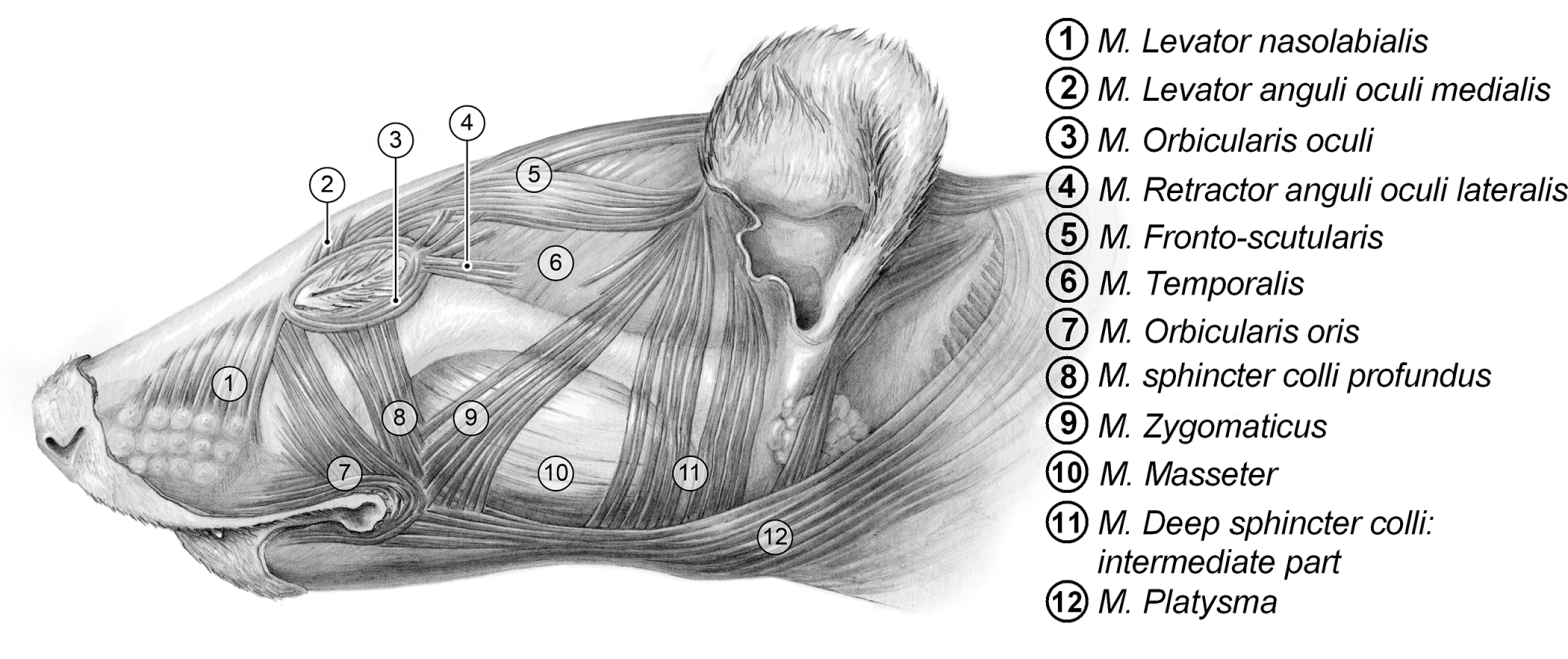
Ferret facial anatomy. Skin and superficial muscles were removed during dissection, drawing made by R. van Deijk.

The detailed anatomical drawing of the ferrets’ facial muscles is shown in Fig 2. The name, origin, insertion and action of these muscles are listed in Table 2.

**Table 2:** Name, origin, insertion and action of the facial muscles of the ferret [30].

Name	Origin	Insertion	Action
*M*. *Levator nasolabialis*	Just off the nose bridge midline, start at eye-level	Side of nose/front of upper lip	Lifts upper lip and wrinkles skin of the nose
*M*. *Levator anguli oculi medialis*	Upper surface of skull, above eye	Top of eye region, merging into the orbicularis oculi	Pulls skin above the eye upward, rearward and slightly inward
*M*. *Orbicularis oculi*	Surrounding the eyes	Inner end attaches to the skull	Closes the eyelid, primarily by depressing the upper eyelid
*M*. *Retractor anguli oculi lateralis*	Side of head to rear of the eye	Outer (rear) corner of eye region	Pulls the corner of the eye caudally
*M*. *Fronto-scutularis*	Lateral eye corner	Scutiform cartilage	Draws the ear cranial
*M*. *Temporalis*	Upper rear part of skull	Top of upward projection of lower jaw	Closes the mouth by lifting and pulling back the lower jaw
*M*. *Orbicularis oris*	Corner of mouth	Into lips as it surrounds mouth	Closes the mouth by tightening the lips
*M*. *sphincter colli profundus*	Near corner of mouth	Into orbicularis oculi, extending upward to lower eyes	Pulls the lower eyelid down, and/or lifts the upper lip
*M*. *Zygomaticus*	Base of ear	Corner of mouth, merging with fibers of orbicularis oris	Draws the corner of the mouth dorso-caudal and external ear ventro-cranial
*M*. *Masseter*	Lower edge of zygomatic arch	Side of lower jaw, into lower and rear edges of lower jaw	Closes mouth by lifting lower jaw
*M*. *Deep sphincter colli*: *intermediate part*	Base of ear	Lateral to masseter muscle, fuses with fascicles of same muscle on opposite side	Turns and pulls ear down
*M*. *Platysma*	Midline on back of the upper neck	Passes over side of lower jaw into corner of mouth, fusing with orbicularis oris	Pulls corner of the mouth backwards

### The Ferret Grimace Scale

**Fig 3:**
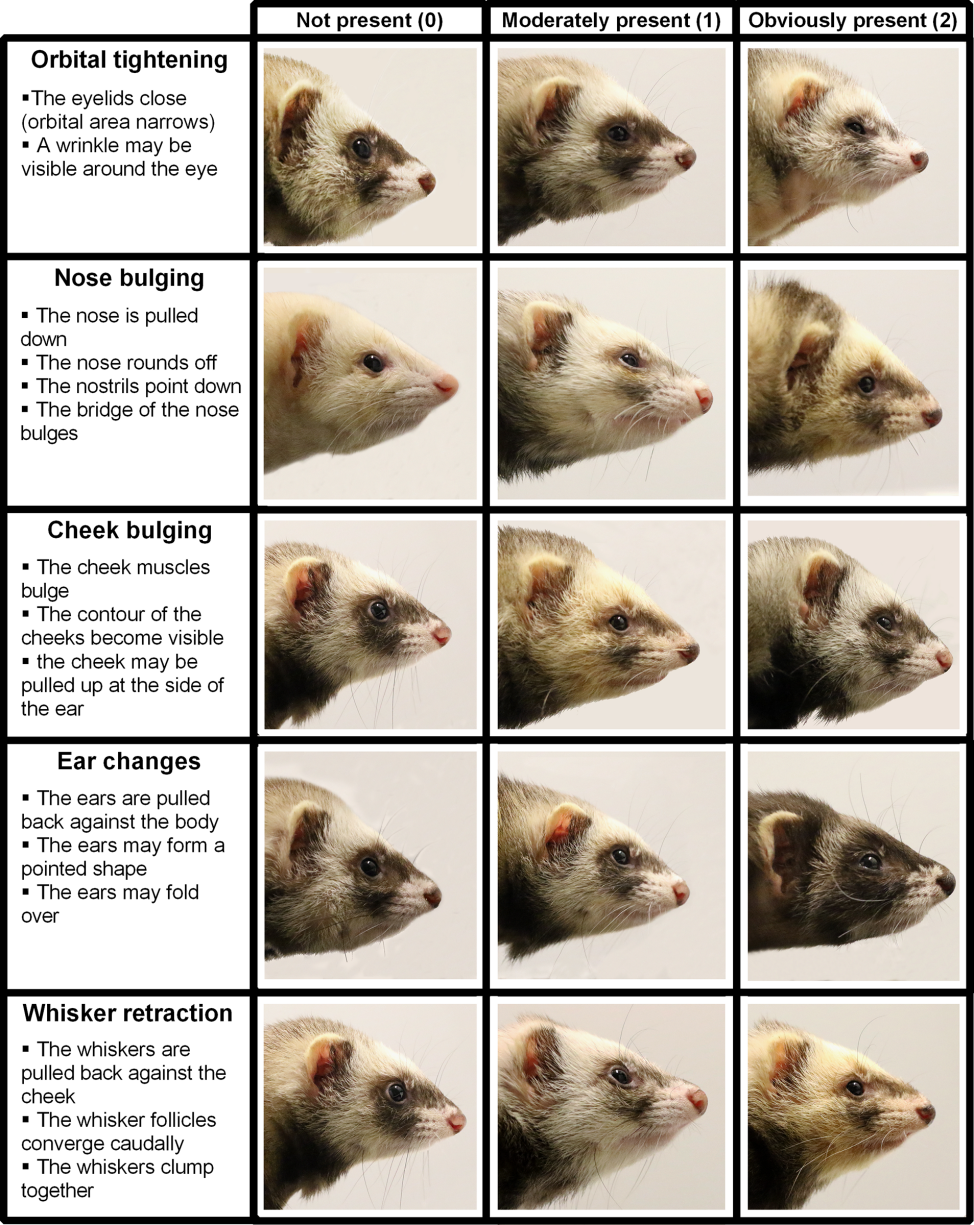
The ferret grimace scale. Photographs visualizing the normal appearance and changes (0 = not present, 1 = moderately present, 2 = obviously present) of the five Action Units that are used in the Ferret Grimace Scale.

Five action units (AUs) were defined from the within-subject comparisons: *orbital tightening*, *nose bulging*, *cheek bulging*, *ear changes* and *whisker retraction*. *Orbital tightening* consists of closing of the eyelids, which can cause a wrinkle to become visible around the eye. *Nose bulging* consists of pulling the nose down, causing the nose to round off, the nostrils to point down instead of straight forward and bulging of the bridge of the nose. *Cheek bulging* occurs due to constriction of the cheek muscles, making the contour of the cheeks become visible. Additionally, the cheek may be pulled up at the side of the ear. *Ear changes* consist of pulling back the ears against the body, possibly forming a pointed shape and folding over. *Whisker retraction* consists of pulling back the whiskers against the cheek, clumping together of the whiskers and caudal convergence of the whisker follicles (Fig 3).

### Missing scores

The number [percentage] of photographs that were scored “I don’t know” (missing scores) by the observers was different between the AUs (LMM: *F*_55_ = 3.108, *P* = 0.022). More specifically, *orbital tightening* (*N* = 8[4%]) had significantly less missing scores than *ear changes* (*N* = 19[9%], *P* = 0.001) and *cheek bulging* (*N* = 15[7%], *P* = 0.027). The number [percentage] of photographs that were scored “I don’t know” for overall pain by the observers was significantly lower after completing the training module on each of the AUs than before this training (LMM: *F*_22_ = 10.333, p = 0.004, before: *N* = 41[20%], after: *N* = 10[5%]). The number [percentage] of photographs with missing scores for the AUs also varied per ferret (LMM: *F*_18_ = 5.846, *P*<0.001). More specifically, two of the three long-haired (angora-like ferrets) had significantly more missing scores for the AUs than the other ferrets (*N* = 10[13%] versus *N* = 5[6%], *P*<0.001 for both ferrets). (Table 3)

**Table 3:** Number and percentage of pictures (*N* = 209) that were scored ‘I not to know’ for *orbital tightening*, *nose bulging*, *cheek bulging*, *ear changes*, *whisker retraction* and overall pain pre- and post-training by 11 observers of 19 ferret faces at different time points (T_B2_, T_B5_, T_2_, T_5_, T_P2_ and T_P5_).

Photographs with missing scores (of 209)														
	Orbital tightening		Nose bulging		Cheek bulging		Ear changes		Whisker retraction		Pre-training		Post-training	
Time	*N*	%	*N*	%	*N*	%	*N*	%	*N*	%	*N*	%	*N*	%
**T**_**B2**_	6	3%	12	6%	14	7%	10	5%	11	5%	42	20%	11	5%
**T**_**B5**_	10	5%	8	4%	19	9%	23	11%	16	8%	46	22%	4	2%
**T**_**2**_	7	3%	9	4%	14	7%	20	10%	14	7%	45	22%	10	5%
**T**_**5**_	5	2%	17	8%	15	7%	17	8%	12	6%	27	13%	8	4%
**T**_**P2**_	7	3%	18	9%	12	6%	18	9%	16	8%	41	20%	12	6%
**T**_**P5**_	12	6%	14	7%	15	7%	23	11%	15	7%	46	22%	14	7%

### Intra- and interrater agreement

The inter-observer agreement for all AUs and for overall pain score were excellent: *orbital tightening*: ICC = 0.97 (*F*_79,790 =_ 29.505, *P*<0.001), *nose bulging*: ICC = 0.85 (*F*_67,670_ = 6.514, *P*<0.001), *cheek bulging*: ICC = 0.86 (*F*_63,630_ = 7.038, *P*<0.001), *ear changes*: ICC = 0.88 (*F*_55,550_ = 8.443, *P*<0.001), *whisker retraction*: ICC = 0.88 (*F*_54,540_ = 8.321, *P*<0.001), overall pain pre-training: ICC = 0.89 (*F*_5,50_ = 10.882, *P*<0.001), overall pain post-training: ICC = 0.89 (*F*_73,730_ = 8.503, *P*<0.001). The intra-observer agreement between overall pain score pre- and post-training was good: ICC = 0.67 (*F*_968_ = 3.017, *P*<0.001).

### AU-scores over time

There were differences in baseline scores for *orbital tightening* (CLMM: *LR* = 210.9036, *P*<0.0001), *nose bulging* (CLMM: *LR* = 220.8676, *P*<0.0001), *ear changes* (CLMM: *LR* = 180.4370, *P*<0.0001) and *whisker retraction* (CLMM: *LR* = 217.2884, *P*<0.0001). More specifically, the scores at baseline for *orbital tightening* and *whisker retraction* were significantly lower at T_B5_ than at T_B2_ (*P* = 0.0024 and *P* = 0.0354, respectively). The scores for *nose bulging* and *ear changes* were significantly higher at T_B5_ than at T_B2_ (*P* = 0.0024 and *P* = 0.0162, respectively).

**Fig 4:**
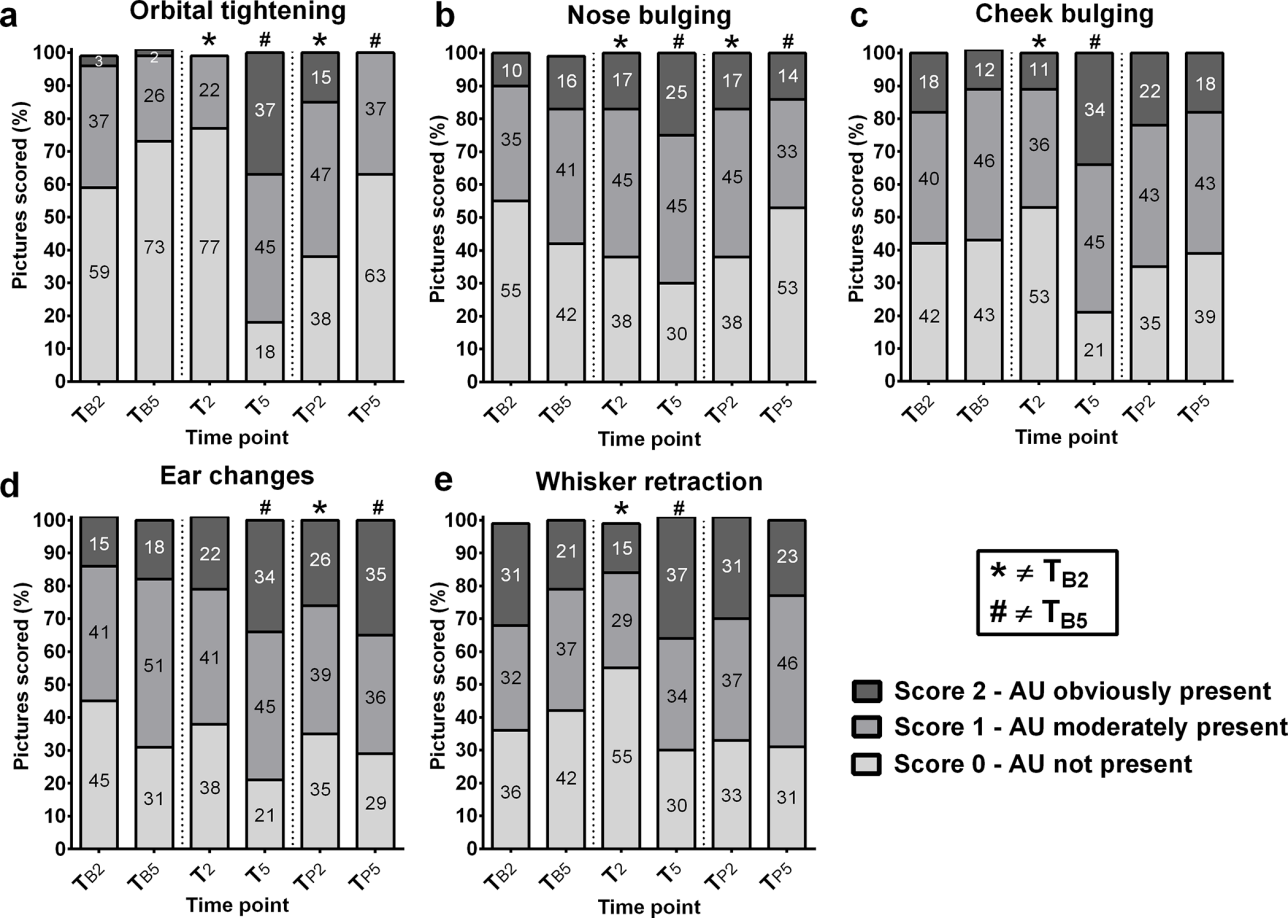
Average percentage of ferrets (*N* = 19) that were assigned score 0, 1, and 2 for each of the AUs in the FGS by 11 observers at different time points. a) *orbital tightening*, b) *nose bulging*, c) *cheek bulging*, d) *ear changes* and e) *whisker retraction* on six time points relative to the time of surgery (T_0_), * = score is significantly different from score on T_B2_, # = score is significantly different from score on T_B5_ with p<0.05 (11 observers, 19 ferrets).

When evaluating the AU *orbital tightening*, time was found to exert a significant effect on the score assigned by the observers (CLMM: *LR* = 210.9036, *P*<0.0001). More specifically, the post-surgery scores at T_2_ were significantly lower than at T_B2_ (*P*<0.0001). Additionally, the post-surgery scores at T_5_, T_P2_ and T_P5_ were significantly higher than their respective baseline scores (T_B5_: *P*<0.0001, T_B2_: *P*<0.0001 and T_B5_: *P* = 0.0428 respectively)(Fig 4A).

Similarly, differences were present in score over time for *nose bulging* (CLMM: *LR* = 220.8676, *P*<0.0001), *cheek bulging* (CLMM: *LR* = 232.0036, *P*<0.0001), *ear changes* (CLMM: *LR* = 180.4370, *P*<0.0001), and *whisker retraction* (CLMM: *LR* = 217.2884, *P*<0.0001). More specifically, for *nose bulging* the post-surgery scores at T_2_, T_5_ and T_P2_ were significantly higher than their respective baseline scores (T_B2_: *P* = 0.0003, T_B5_: *P* = 0.0048, T_B2_: *P* = 0.0002 respectively), whereas the post-surgery scores at T_P5_ were significantly lower than at baseline (T_B5_: *P* = 0.0393)(Fig 4B). For *ear changes*, post-surgery scores at T_5_, T_P2_ and T_P5_ were significantly higher than their respective baseline scores (T_B5_: *P* = 0.0004, T_B2_: *P* = 0.0039, T_B5_: *P* = 0.0107 respectively)(Fig 4D). For *cheek bulging* and *whisker retraction*, only the post-surgery scores at T_2_ and T_5_ differed significantly from their respective baselines, with the post-surgery scores at T_2_ being significantly lower than at baseline (T_B2_: *P* = 0.0029 and *P*<0.0001 for *cheek bulging* and *whisker retraction*, respectively) and the post-surgery scores at T_5_ being significantly higher than at baseline (T_B5_: *P*<0.0001 and *P* = 0.0003 for *cheek bulging* and *whisker retraction*, respectively)(Fig 4C and 4E).

### Weight, sensitivity, specificity and accuracy

**Fig 5:**
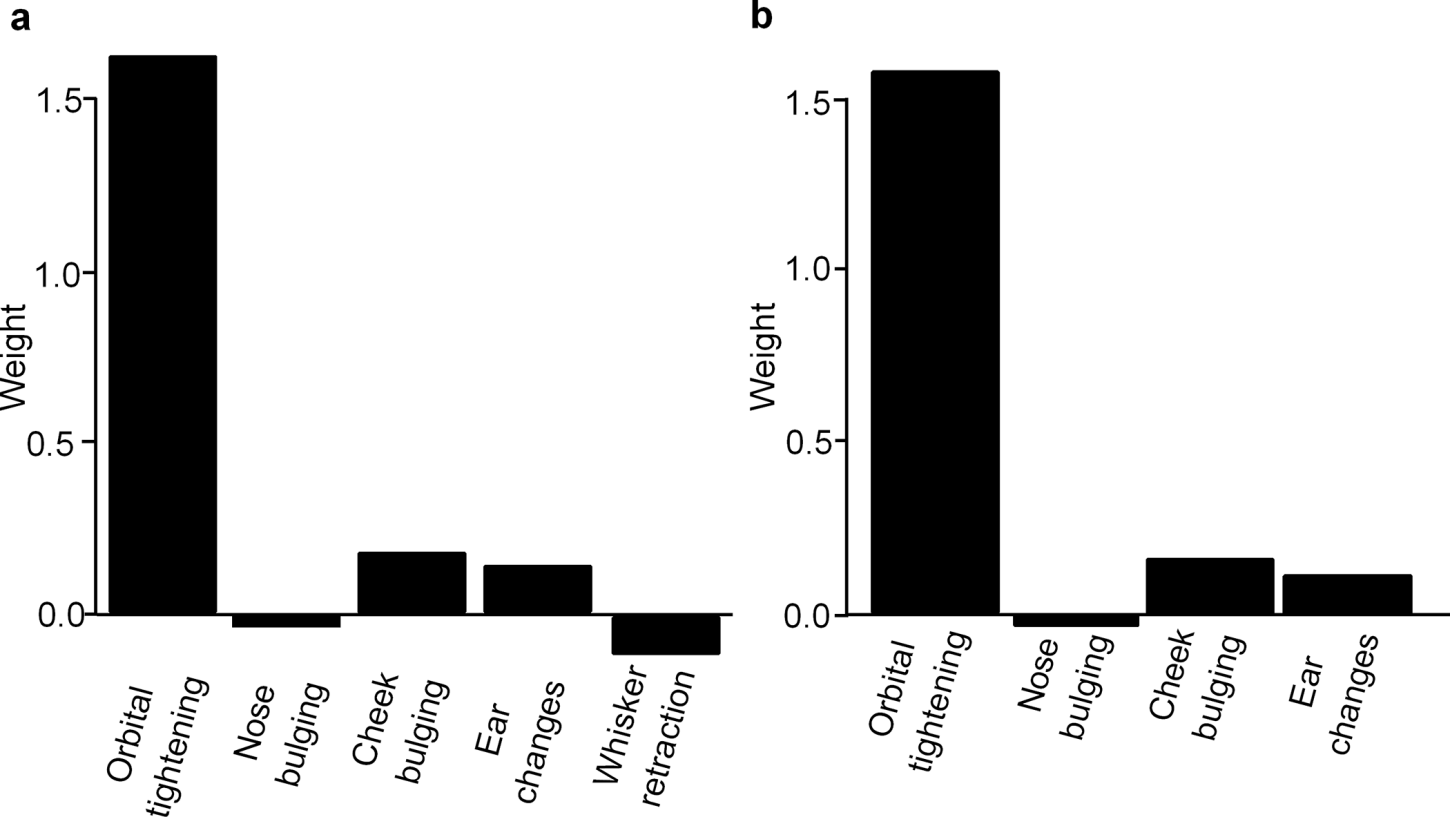
Optimal weights of each AU. Obtained by performing the LDA on the observer scores for *orbital tightening*, *nose bulging*, *cheek bulging*, *ear changes and whisker retraction*. a) using all AUs, b) without *whisker retraction*.

From the LDA, it was clear that *whisker retraction* had a negative weight, close to zero (Fig 5A). Therefore, *whisker retraction* was removed from the model and the weights of the other AUs were re-estimated by performing another LDA. In this model, like the first model, *cheek bulging*, *nose bulging* and *ear changes* had weights close to zero (Fig 5B).

Using the optimal weights of the AUs obtained by performing the LDA, the sensitivity, specificity and accuracy of the observer scores were calculated 1) using all five AUs, 2) using the FGS without *whisker retraction* and 3) using only *orbital tightening*. In all these three options, the score 1 for the *orbital tightening* AU was the cut-off value for which a photograph was classified as taken at T_B5_ (score of *orbital tightening* below 1, i.e. score of *orbital tightening* equal to 0) or at T_5_ (score of *orbital tightening* equal to 1 or 2). The sensitivity (85%) and specificity (74%) were equal for all three options. The highest accuracy (80%) was achieved using the FGS without *whisker retraction* and using only *orbital tightening*.

## Discussion

With this study, we aimed to compose a Ferret Grimace Scale (FGS) using action units (AUs) that were based on the facial musculature of ferrets. Additionally, we wanted to evaluate whether the descriptions of the AUs were clear and aided observers in assigning an overall pain score to photographs that were taken before and after telemetry probe implantation surgery. Furthermore, we aimed to determine whether the AUs could be used to distinguish ferrets before and after this surgery. Finally, we aimed to make an informed decision on which AUs could be included in a final FGS in their current form and which AUs should be re-evaluated by assessing the weight, sensitivity, specificity and accuracy of the AUs comprising the scale.

### Facial musculature and the FGS

The ferrets’ facial musculature is very similar to that of mice and rats [31,32], containing all the necessary muscles to show different facial expressions. Therefore, it is not surprising that the potential AUs that were identified for the ferrets (*orbital tightening*, *nose bulging*, *cheek bulging*, *ear changes* and *whisker retraction*) are very similar to those in the other grimace scales, e.g. the mouse, rat and rabbit grimace scale [11–13]. Comparing the different grimace scales, the similarities are striking, raising the question whether it is necessary to develop a grimace scale for every animal species or whether we can reduce the amount of animals that are used in research by making a generalized vertebrate grimace scale. However, there are also species differences in the AUs of established Grimace Scales. For example, where rats and rabbits flatten their nose/cheek when in pain, mice (and ferrets) bulge their nose/cheek [11–13]. The only AU that seems to appear in the same form in all species is *orbital tightening*.

### Missing values and agreement

Based on the number of missing values and the inter-observer reliability, the observers seemed to find the action unit (AU) *orbital tightening* the most clearly described and/or the most recognisable. The observers seemed to find the AUs *ear changes* and *cheek bulging* the least clear, as there were more missing values for these AUs. Therefore, it would be worthwhile to re-evaluate the descriptions of at least *ear changes* and *cheek bulging*, but preferably also *nose bulging* and *whisker retraction*.

The ability of observers to assign a score for the AUs could have potentially been influenced by the type of coat the ferret had, which seemed to be more difficult in the longer-haired ferrets. This has not previously been reported, but is not surprising, as the long hair might have obscured muscle tension and/or ear position. This should therefore be taken into account when applying the FGS and when selecting ferrets as animal models (e.g. selecting only short-haired ferrets).

The observers scored the overall pain with excellent and good consistency (inter- and intra-observer, respectively), indicating that the observers agreed on which score to assign to a photograph, as well as that an observers’ first (pre-training) assessment was equal to the second (post-training assessment). The intra-observer reliability might have lower agreement due to a training effect, i.e. as the observers have been trained to look more closely at the ferrets’ faces in the survey-parts concerning the AUs. The observers did appear more confident to assign an overall pain score to the photographs after seeing the examples of and having scored the photographs on the AUs, as there were less missing values for the overall pain scores post-training than pre-training. This indicates that systematically looking at the ferrets’ faces using the AUs can aid in formulating an overall pain score.

### Scores over time

The baseline FGS scores at T_B2_ were significantly different from the baseline scores at T_B5_ for the AUs *orbital tightening*, *whisker retraction*, *ear changes* and *nose bulging*, which suggests that the time of day influences the facial expression of the ferrets. These differences in AU-scores might possibly be explained by the polycyclic sleep-wake cycle of ferrets: ferrets sleep in bouts of approximately three hours [33], which is exactly the window between the two baseline measurements. As the ferrets were used to being disturbed in the morning (around T_B2_), but not in the afternoon (around T_B5_) and they are known to adapt their sleep-wake cycle to human activity, the ferrets might have been awake around T_B2_ and just woke up around T_B5_. This is supported by asleep, waking or sedated animals showing false positives with other grimace scales (i.e. mice [11] and rats [12]). Using more than two measurements on one day and using a smaller inter-measurement period could provide more information on the background of these differences in scores in ferrets.

Two hours after the surgery (T_2_), the scores for *orbital tightening*, *nose bulging and cheek bulging* were significantly lower than baseline (T_B2_), indicating that there might still have been a carry-over sedative or analgesic effect of ketamine (as the effects of dexmedetomidine were reversed using atipamezole). Studies in mink reported recovery within 60 or even 15 minutes after reversal with the chosen anaesthetic regime [24,25]. However, possible carry-over effects of ketamine have not been addressed in these studies. Therefore, the changes observed in the ferrets’ facial expressions two hours after surgery might be a reflection of recovery from the anaesthesia. This hypothesis is supported by the observation that some ferrets were repeatedly yawning during handling at this time point, which was not seen at other time points (but would be expected when yawning was expressed as a normal behaviour when waking up). It has been shown that isoflurane, and not buprenorphine, increased the mouse grimace score in male DBA/2 mice [34], which underlines the need to carefully choose the post-surgery time to ensure the animals are no longer experiencing the major sedative effects of anaesthesia or the anaesthetic regimes for grimace scale research. Additionally, it should be taken into account that the ferrets received equal amounts of anaesthetics, while their bodyweights differed. Some of the ferrets might therefore have experienced stronger/longer lasting sedation/analgesia than others.

The scores for all AUs and overall pain scores peaked five hours after surgery (T_5_), which is in line with the results found in other grimace scale studies (e.g. the rat grimace scale [12]). This high FGS-score suggests that the ferrets were experiencing pain at that moment, supported by the observation that the ferrets showed repeating bouts of pronounced shivering when they were handled at this time point. The ferrets did not show shivering during handling at T_2_ or at any other time point, which makes it unlikely that the shivering was caused by hypothermia, stress or as a result of recovery from the anaesthesia. However, due to the lack of control animals receiving analgesics, clear conclusions cannot be drawn on the pain-state of the ferrets. Follow-up studies using multiple analgesic schedules should be performed to confirm this as this will further confirm that the changes in the FGS observed here are pain-related. In these follow-up studies, it should be taken into account that there might be individual and sex differences in pain perception and therefore in pain expression.

The scores of the photographs taken the day after surgery (T_P2_ and T_P5_) were significantly lower than the scores at T_5_, but were still higher than baseline scores for *orbital tightening*, *nose bulging* and *ear changes*. This could possibly indicate that the chosen time frame for this study was too short to capture the point at which the ferrets’ grimace (and pain-state) was back to baseline. It was unexpected that the ferrets would still show changes in the AUs 26 and/or 29 hours after surgery as the recovery time in rats was shorter, their grimace scores were back to baseline twelve hours after a laparotomy [12]. However, these differences between the species/studies might have occurred due to other pain-related causes, e.g. the size of the incision (relative to the animal) or the method with which the wounds were closed (sutures or tissue glue) or non-pain-related causes, e.g. the anaesthesia that was used.

The decline in AU scores over time might indicate a slow recovery from pain, but again, this should be further confirmed using a control group that receives analgesics. In a follow-up study, it would be informative to measure more than two time points per day to identify the FGS-peak and to take FGS-scores later than 29 hours after surgery, to determine the point at which the AU scores are all back to baseline. Additionally, it would be preferable to include other clinical signs that are considered indicative of pain (physiological and behavioural changes) to further validate this scale.

### Weight, sensitivity, specificity and accuracy

Even though the scores for all AUs increased significantly from baseline to five hours after surgery (T_5_), just the scores for *orbital tightening* were required to achieve a high sensitivity, specificity and accuracy, which is comparable to the findings of other studies [11–14]. Additionally, *orbital tightening* had the least missing values, indicating that it was the most clear/most visible AU for observers. It is not surprising that *orbital tightening* is the most important AU as this is also reported for other grimace scales (e.g. [19]) and humans tend to focus on the eyes of people/animals when assessing emotions such as pain [35,36].

Including the AUs *nose bulging*, *cheek bulging* and *ear changes* alongside *orbital tightening* in the FGS resulted in a similar accuracy of score assignment as *orbital tightening alone*. However, in comparison to *orbital tightening*, the weights assigned to these three AUs in the overall scoring were low, indicating that they contributed only slightly to the overall score. Moreover, many values were missing indicating that observers had difficulty in assigning a score for these three AUs. For the AU *whisker retraction*, a negative weight was even found and inclusion of this AU actually resulted in a lower accuracy, which indicates it might have confused the observers in the pain assessment. However, it should be taken into account that sensitivity, specificity and accuracy were calculated using the assumption (supported by LDA results) that all AUs were absent at T_B5_ and moderately or obviously present at T_5_ in each photograph, which might not have been the case as the FGS is not yet validated. This assumption might have led to an erroneously low or high accuracy. Moreover, since this, to the authors’ knowledge, is the first study to assess the weight of individual AUs, it is currently not possible to compare our results with other grimace scales that incorporate *whisker retraction*, *nose bulging*, *cheek bulging* and *ear changes*, such as those of the mouse, rat and rabbit [11–13]. Nevertheless, our findings suggest that the description of the four AUs that resulted in low or negative scores should be re-evaluated to explore whether clarity and/or visibility can be increased. Additionally, the effects of live scoring, other photograph angles and settings should be explored as this might result in more reliable results for all AUs.

## Conclusion

Overall, the results of this study suggest that the FGS and the AU *orbital tightening* in particular, could be useful for pain assessment of ferrets. The other AUs (*whisker retraction*, *nose bulging*, *cheek bulging* and *ear changes*) should be re-evaluated before they can be included in the FGS. Furthermore, prior to incorporating the FGS in a multifactorial pain assessment protocol, it should be further validated using different painful stimuli, analgesic regimens and measuring more time points.

## Supporting information

S1 Table Pre-training observer scores for overall pain. 0 = no pain, 1 = mild pain, 2 = moderate pain, 3 = severe pain or? = missing.
(XLSX)

S2 Table Observer scores for ear changes. 0 = not present, 1 = moderately present, 2 = obviously present,? = missing.
(XLSX)

S3 Table Observer scores for orbital tightening. 0 = not present, 1 = moderately present, 2 = obviously present,? = missing.
(XLSX)

S4 Table Observer scores for cheek bulging. 0 = not present, 1 = moderately present, 2 = obviously present,? = missing.
(XLSX)

S5 Table Observer scores for whisker retraction. 0 = not present, 1 = moderately present, 2 = obviously present,? = missing.
(XLSX)

S6 Table Observer scores for nose bulging. 0 = not present, 1 = moderately present, 2 = obviously present,? = missing.
(XLSX)

S7 Table Post-training observer scores for overall pain. 0 = no pain, 1 = mild pain, 2 = moderate pain, 3 = severe pain or? = missing.
(XLSX)
